# Bioinformatics analysis and experimental validation of tumorigenic role of PPIA in gastric cancer

**DOI:** 10.1038/s41598-023-46508-y

**Published:** 2023-11-05

**Authors:** Jichao Liu, Yanjun Wang, Zhiwei Zhao, Yanhui Ge

**Affiliations:** 1 Department of Gastrointestinal Surgery, The First Affiliated Hospital of Zhengzhou University, Zhengzhou University, Henan, China; 2 Department of Vascular Surgery, The First Affiliated Hospital of Zhengzhou University, Zhengzhou University, Henan, China; 3 Department of Pharmacy, The First Affiliated Hospital of Zhengzhou University, Zhengzhou University, Henan, China

**Keywords:** Cancer, Genetics, Immunology, Molecular biology, Biomarkers, Diseases, Gastroenterology

## Abstract

Gastric cancer (GC) is a malignant tumor with high incidence rate and mortality. Due to the lack of effective diagnostic indicators, most patients are diagnosed in late stage and have a poor prognosis. An increasing number of studies have proved that Peptidylprolyl isomerase A (PPIA) can play an oncogene role in various cancer types. However, the precise mechanism of PPIA in GC is still unclear. Herein, we analyzed the mRNA levels of PPIA in pan-cancer. The prognostic value of PPIA on GC was also evaluated using multiple databases. Additionally, the relationship between PPIA expression and clinical factors in GC was also examined. We further confirmed that PPIA expression was not affected by genetic alteration and DNA methylation. Moreover, the upstream regulator miRNA and lncRNA of PPIA were identified, which suggested that LINC10232/miRNA-204-5p/PPIA axis might act as a potential biological pathway in GC. Finally, this study revealed that PPIA was negatively correlated with immune checkpoint expression, immune cell biomarkers, and immune cell infiltration in GC.

## Introduction

Gastric cancer (GC) is the fifth most frequent cancer type and the fourth cause of cancer mortality in the world^1^. In 2020, an estimated over one million new cases of GC and 769,000 deaths worldwide according to the GLOBOCAN 2020 database. Moreover, Eastern Asia had the highest incidence rate for GC. However, China accounts for 43.9% of the new GC cases and 48.6% of the cancer deaths worldwide^2^. In China, the 5-year survival of GC is 35.1%^3^. Due to the lack of early diagnosis biomarkers, most of the GC patients have entered the clinical medical-advanced stage and have relatively poor prognosis^4^. The incidence rate of GC has been kept high in China, which severely affects human life and health. Thus, it is of great importance to explore effective targets for the treatment, diagnosis and prevention of GC.

Recently, the rapid development of molecular bioinformatics offers a new mentality for the diagnosis and treatment of cancer^5–7^. Various biomedical public databases, such as The Cancer Genome Atlas (TCGA), provide cancer researchers with massive genomic data and associated clinical data^8–10^. The public databases were utilized to discover biomarkers and biological mechanism of tumor origination, development and metastasis by mining meaningful genomic changes^11^. Next-generation sequencing technology (NGS) is widely used in the study of tumor pathogenesis, diagnosis, treatment and prognosis analysis, and has made a series of major breakthroughs^12,13^. The development of precision medicine characterized by genomic data and individualized medicine may affect clinical practice and improve the survival rate of cancer patients^14,15^.

PPIA encodes a member of the peptidyl-prolyl cis–trans isomerase (PPIase) family, which accelerates protein folding by catalyzing the cis–trans isomerization of proline imidic peptide bonds in oligopeptides. Moreover, PPIA participates in the modulation of various biological processes, including apoptosis, inflammation, transcription and intracellular signaling^16–19^. Previous studies suggest that the extracellular fractions of PPIA are potent pro-inflammatory mediators and PPIA is associated with a variety of inflammatory diseases^20^. Recent studies indicate that PPIA also plays a pivotal role in the development of human cancers^16^, including colon cancer^21^, hepatocellular carcinoma (HCC)^22^, multiple myeloma^23^, lung adenocarcinoma^24^ and GC^25^. For example, Gu et al.^26^ showed that PPIA was overexpressed in HCC and involved in the occurrence of HCC by modulating tumor immunity and mRNA metabolic process. Davra et al.^27^ demonstrated that PPIA was involved in host antitumor immune evasion, tumor metastasis, and cell migration in breast cancer. Although some studies have proved that PPIA could act as an oncogene and biomarker in various cancer types, the precise mechanisms of PPIA in GC are poorly understood.

Herein, the expression levels of PPIA in pan-cancer were analyzed. We focused our analysis on the expression and prognostic values of PPIA and investigated the clinical correlation of PPIA expression in GC. Next, we performed functional enrichment, genetic alteration and DNA methylation analyses of PPIA in GC. We also identified the upstream regulator ncRNAs of PPIA in GC. Finally, we confirmed the function of PPIA in the immune system of GC. To sum up, our results indicated that ncRNAs-regulated overexpression of PPIA was associated with immune cell infiltration (ICI) and poor prognosis in GC.

## Results

### Expression of PPIA in pan-cancer and prognostic value of PPIA in GC

**Figure 1:**
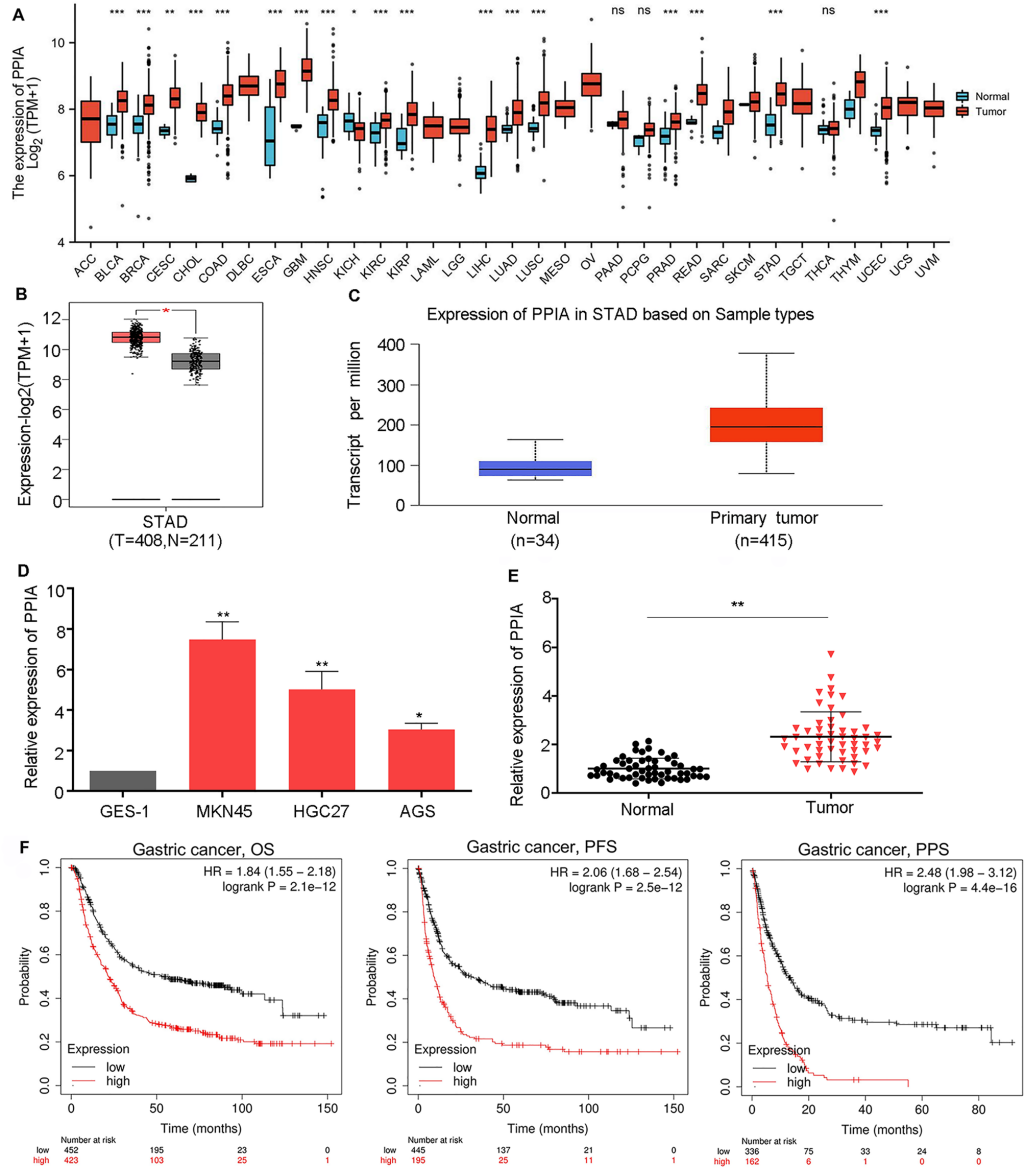
Expression of PPIA in pan-cancer and prognostic significance of PPIA in GC. (**A**) Expression levels of PPIA in 33 human cancer types according to TCGA database. (**B**) Expression analysis of PPIA in GC using GEPIA database. (**C**) Expression analysis of PPIA in GC using UALCAN database. (**D**) The relative expression of PPIA in GES1, MKN45, HGC27 and AGS cells based on qRT-PCR. (**E**) The relative expression of PPIA in 53 paired GC tissues and corresponding normal tissues. (**F**) Prognostic analysis of PPIA in GC using K-M plotter. *p < 0.05; **p < 0.01; ***p < 0.001; *ns* the difference is not statistically significant.

To assess the effects of PPIA on the genesis of human tumor, TCGA database was utilized to detect the mRNA levels of PPIA in 33 types of cancer. The findings demonstrated that the levels of PPIA were upregulated in 17 tumor types including BRCA, BLCA, CHOL, COAD, CESC, ESCA, HNSC, GBM, KIRP, KIRC, LIHC, LUAD, LUSC, READ, PRAD, UCEC and STAD compared to the corresponding normal tissues (Fig. 1A). However, there was no obvious difference of PPIA in DLBC, ACC, LGG, LAML, OV, MESO, TGCT, UVM and UCS. We also demonstrated that PPIA was downregulated in KICH. TIMER database was also used to validate PPIA expression in multiple tumor types. Notably, the high expression levels of PPIA could be observed in BRCA, BLCA, COAD, CHOL, CESC, HNSC, GBM, ESCA, KIRP, KIRC, LUSC, LUAD, LIHC, PRAD, PCPG, STAD, UCEC and READ (Supplementary Fig. 1). Taken together, PPIA was increased in BRCA, BLCA, COAD, CHOL, HNSC, ESCA, KIRP, KIRC, LUSC, LUAD, LIHC, READ, PRAD, UCEC and STAD. This suggests that PPIA may act as an important oncogene in 15 tumor types. Due to a lack of research on this topic, we further evaluated the difference in PPIA expression between the tumor and normal tissues in GC. As shown in Fig. 1B, C, based on Gene Expression Profiling Interactive Analysis (GEPIA) and UALCAN: A Portal for Facilitating Tumor Subgroup Gene Expression and Survival Analyses (UALCAN) databases, PPIA was upregulated in GC compared to normal controls. To validate this result, we detected PPIA expression in a normal gastric mucomembrane cell line (GES-1) and three GC cell lines (MKN45, HGC27 and AGS) and the result showed that PPIA was significantly overexpressed in all GC cell lines compared with GES-1 (Fig. 1D). Moreover, we also explore the expression level of PPIA in 53 pairs GC tissues and corresponding normal tissues. The data suggested that PPIA was obviously upregulated in GC tissues compared to normal tissues (Fig. 1E). Next, we examined the prognostic value of PPIA in GC using K-M plotter. As displayed in Fig. 1F, GC patients with high PPIA expression exhibited poor overall survival (OS), progress free survival (PFS) and post progression survival (PPS). This result suggests that overexpression of PPIA can predict poor prognosis in GC patients.

### The relevance between PPIA expression and clinical parameters in GC patients

**Figure 2:**
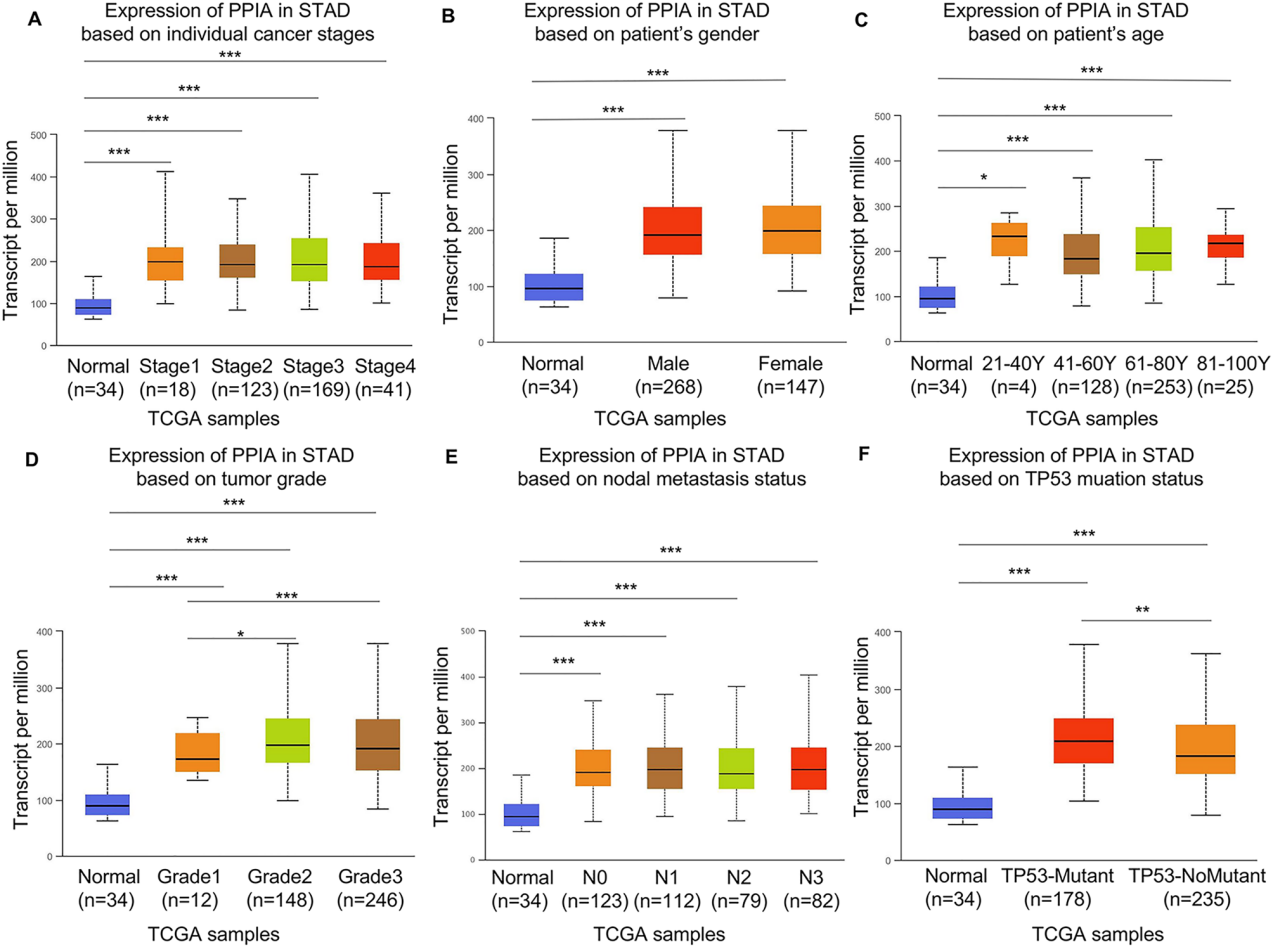
The association between PPIA expression and different clinical parameters in GC according to UALCAN database. Correlation analysis including cancer stages (**A**), gender (**B**), age (**C**), tumor grade (**D**), nodal metastasis status (**E**) and TP53 mutation status (**F**). *p < 0.05; **p < 0.01; ***p < 0.001.

The UALCAN database was utilized to evaluate the relationship between PPIA expression and different clinical parameters. The findings demonstrated that elevated expression of PPIA was detected in GC tissues compared to the corresponding normal tissues. However, there were no significant difference between different cancer stages (Fig. 2A). Similarly, an increased expression of PPIA was also observed in both male and female GC specimens compared to normal controls and no difference were detected between male and female (Fig. 2B). Then, PPIA expression was highly upregulated in GC specimens based on different age groups (21–40, 41–60, 61–80 and 81–100 years), we did not observed differences among different age groups (Fig. 2C). According to tumor grade, an increased expression level of PPIA was observed in gastric patients. Moreover, there were significant differences between Grade 1 and Grade 2 (*p* < 0.05), Grade 1 and Grade 3 (*p* < 0.0001) (Fig. 2D). Furthermore, the upregulation of PPIA in GC was associated with nodal metastasis status, and patients with N0, N1, N2 and N3 displayed a higher PPIA expression compared to normal controls. However, no significant difference was found in different nodal metastasis groups (Fig. 2E). Moreover, high expression of PPIA was also observed in GC patients with TP53 wild-type and mutant compared to normal controls. Significant difference also detected between TP53 wild-type and mutant group (Fig. 2F).

Regarding patient’s race, high expression of PPIA was observed in Caucasians, African-American, and Asian (Supplementary Fig. 2A). PPIA was also remarkably upregulated in GC patients with and without *Helicobacter pylori* infection (Supplementary Fig. 2B). All statistics detailed in Supplementary Table 1. Our data showed that high PPIA expression was associated with poor histological grade and TP53 mutation.

### Functional enrichment analysis of PPIA in GC based on TCGA database

**Figure 3:**
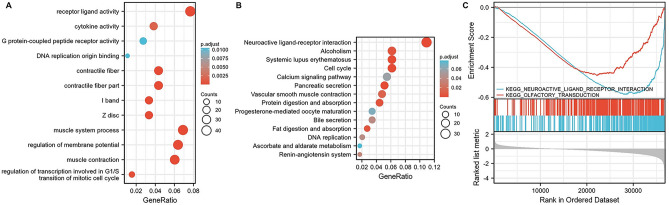
Functional enrichment analysis of PPIA in GC. (**A**) GO analysis of FAM72A-D. (**B**) KEGG pathway analysis of PPIA in GC (www.kegg.jp/kegg/kegg1.html). (**C**) Two pathways related to PPIA were significantly enriched in GC.

Differentially expressed genes (DEGs) associated with PPIA in GC were recognized using TCGA database. The top 1000 differential genes related to PPIA were chosen for Gene Ontology (GO) and Kyoto Encyclopedia of Genes and Genomes (KEGG) pathway analysis. The top GO enrichment items were receptor ligand activity, G protein-coupled peptide receptor activity, cytokine activity, DNA replication origin binding, contractile fiber, I band, Z disc, muscle system process, regulation of membrane potential, muscle contraction, and transcriptional regulation involve in G1/S transition of mitotic cell cycle (Fig. 3A). The top KEGG pathways of PPIA were systemic lupus erythematosus, alcoholism, neuroactive ligand-receptor interaction, calcium signaling pathway, cell cycle, pancreatic secretion, vascular smooth muscle contraction, protein digestion and absorption, progesterone-mediated oocyte maturaction, bile secretion, fat digestion and absorption, DNA replication, ascorbate and aldarate metabolism, and renin-angiotensin system (Fig. 3B). We also implemented Gene Set Enrichment Analysis (GSEA) analysis to confirm the key pathways correlated with PPIA (Fig. 3B). The results showed that olfactory transduction and neuroactive ligand-receptor interaction were the most significantly enriched pathways (Fig. 3C).

### Genetic alteration and DNA methylation analysis of PPIA

We explored genetic alteration status of PPIA in pan-cancer according to TCGA database, and there were four types of genetic alteration patterns (deep deletion, amplification, structural variant and mutation) in different tumor samples. Amplification displayed the highest alteration frequency in most tumor types and amplification was the only alteration type in GC samples (Supplementary Fig. 3A). Missense mutation was the main type of genetic alteration for PPIA (Supplementary Fig. 33B); however, we did not find any PPIA mutation sites in GC cases. To assess the correlation between PPIA genetic alteration and survival prognosis in GC patients, cBioPortal database was used to perform the prognostic analysis (Supplementary Fig. 3C). The prognosis of PPIA alteration group showed better prognosis in disease-free survival (DFS) (*p* = 0.0309), but not disease-specific survival (DSS) (*p* = 0.312), progression-free survival (*p* = 0.623) and OS (*p* = 0.839). To further evaluate the mechanism of PPIA overexpression in GC, the methylation level of PPIA in GC samples (n = 395) and adjacent normal tissues was analyzed using UALCAN and DiseaseMeth version 3.0 database. However, no relationship between DNA methylation and expression of PPIA was observed (Supplementary Figs. 4A and 4B). This result suggests that the genetic alteration and DNA methylation of PPIA play a minor role in gastric carcinogenesis.

### The upstream miRNAs of PPIA

**Figure 4:**
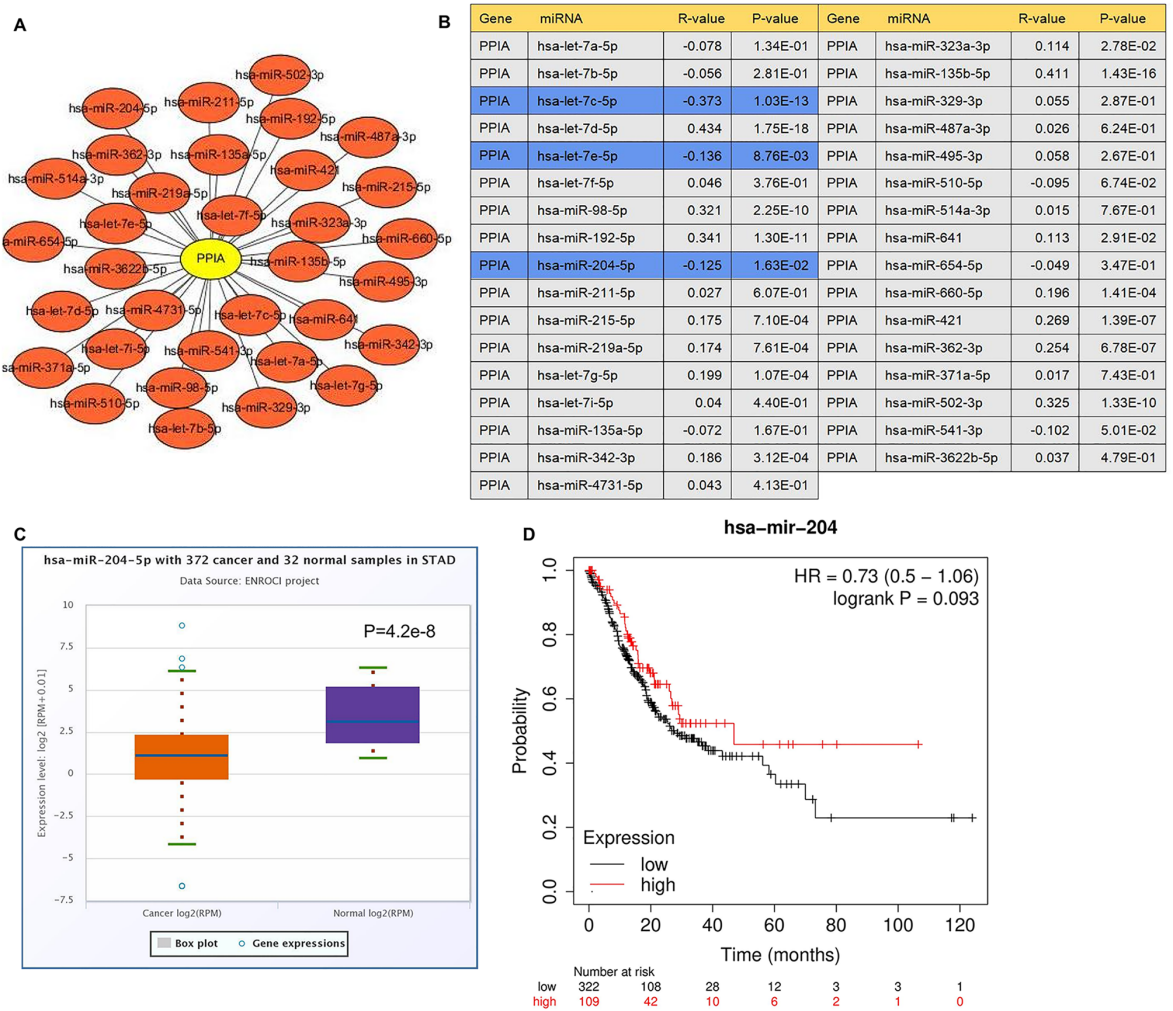
miRNA-204-5p act as an upstream miRNA of PPIA in GC. (**A**) miRNA-PPIA interaction networks constructed via Cytoscape_3.2.1 (https://cytoscape.org/). (**B**) The relationship between potential upstream miRNAs and PPIA in GC. (**C**) Expression of miRNA-204-5p in GC confirmed using starBase 3.0 database. (**D**) Survival analysis of miRNA-204-5p in GC analyzed using K-M plotter.

It is well known that ncRNAs play an essential role in the regulation of gene expression. To confirm whether PPIA was modulated by ncRNAs, starBase 3.0 database was applied to estimate potentially upstream miRNAs of PPIA, we found a total of 33 unique miRNAs associated with PPIA, and cytoscape software was employed to draw miRNA-PPIA regulatory network (Fig. 4A). Accumulating evidence implicated miRNAs could negatively regulate the expression of target genes^28,29^. In this study, let-7c-5p, let-7e-5p and miRNA-204-5p were obviously negatively correlated with PPIA, and were selected as candidate upstream miRNAs of PPIA in GC (Fig. 4B). As shown in Fig. 4C, Supplementary Figs. 5A and 5B, let-7c-5p, let-7e-5p and miRNA-204-5p were significantly downregulated in GC. We also performed the prognosis analysis of let-7c-5p, let-7e-5p and miRNA-204-5p in GC (Fig. 4D, Supplementary Figs. 5C and 5D). It was observed that only patients with miRNA-204-5p overexpression had a good prognosis, although the results were not statistically significant. Finally, miRNA-204-5p was considered as the most appropriate regulatory miRNA of PPIA in GC.

### The upstream lncRNAs of miRNA-204-5p

**Figure 5:**
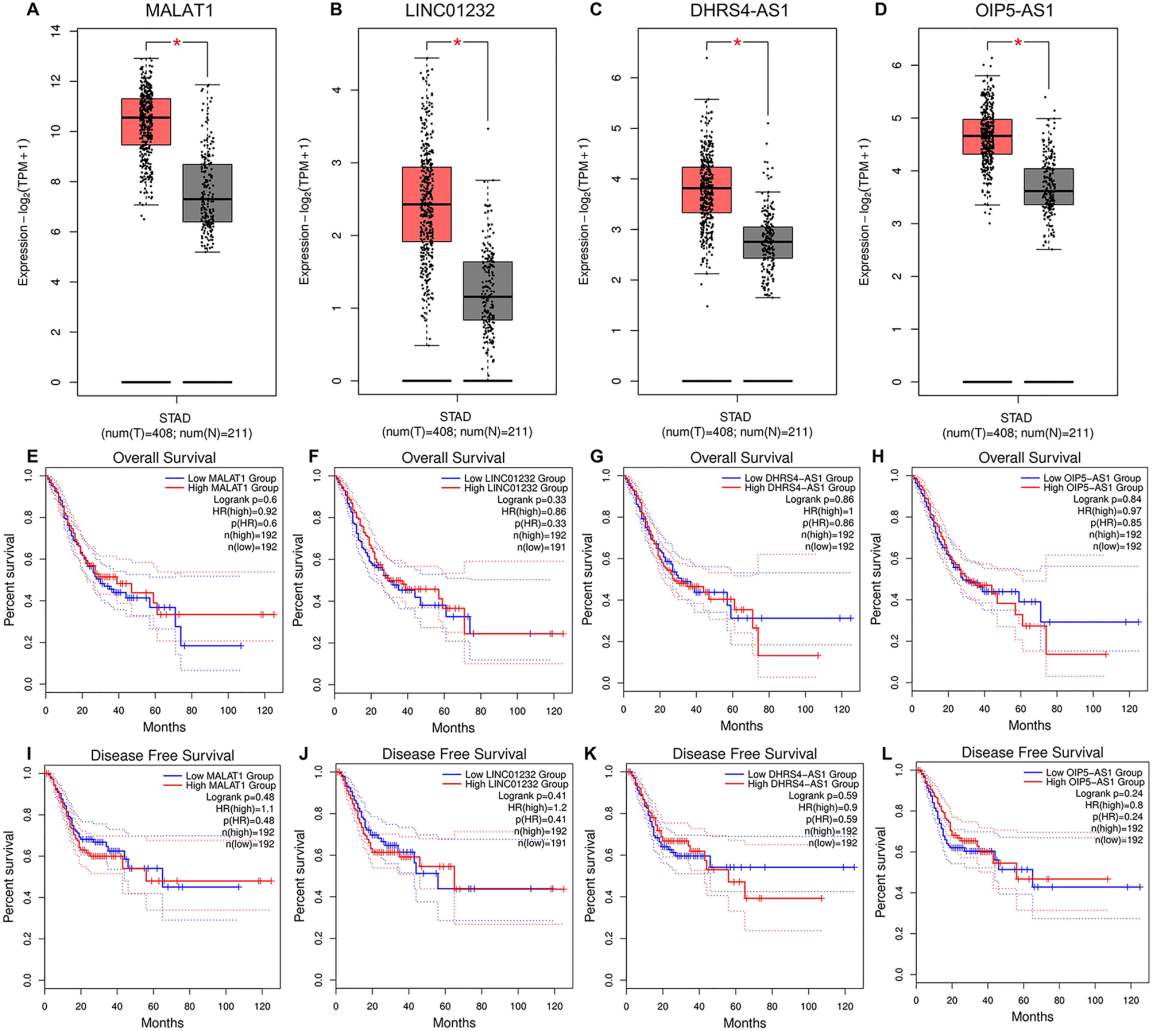
Identification and prognostic evaluation of the upstream lncRNAs of miRNA-204-5p. The expression analysis of MALAT1 (**A**), LINC01232 (**B**), DHRS4-AS1 (**C**) and OIP5-AS1 (**D**) in GC according to TCGA database. The overall survival analysis of MALAT1 (**E**), LINC01232 (**F**), DHRS4-AS1 (**G**) and OIP5-AS1 (**H**) in GC using GEPIA database. The disease-free survival analysis of MALAT1 (**I**), LINC01232 (**J**), DHRS4-AS1 (**K**) and OIP5-AS1 (**L**) in GC using GEPIA database. *p < 0.05.

The starBase 3.0 database was utilized to analyze the upstream lncRNAs of miRNA-204-5p in GC. Supplementary Fig. 6 illustrates a total of 44 lncRNAs that related to miRNA-204-5p. GEPIA database was employed to display the expression and survival analyses the 44 upstream lncRNAs correlated with miRNA-204-5p. Notably, only MAlAT1, LINC01232, DHRS4-AS1 and OIP5-AS1 were markedly upregulated in GC compared with normal controls (Fig. 5A–D). However, there were no obvious differences in DFS and OS of these lncRNAs in GC (Fig. 5E–L). Due to the structural similarity between LncRNAs and mRNA, miRNAs might negatively regulate the expression of LncRNAs through a mechanism similar to mRNA, thereby exerting a series of biological effects^30,31^. As shown in Table 1, only LINC01232 was negatively and positively correlated with miRNA-204-5p and PPIA, respectively. This result indicates that LINC01232 may act as a key candidate upstream lncRNA of miRNA-204-5p/PPIA in GC.

**Table 1:** Correlation analysis between lncRNA and miR-204-5p or lncRNA and PPIA in GC performed by starBase database.

lncRNA	miRNA	R value	*p* value
MALAT1	miR-204-5p	0.017	7.41E−01
LINC01232	miR-204-5p	− 0.134^a^	9.74E−03**^a^
DHRS4-AS1	miR-204-5p	− 0.053^a^	3.11E−01
OIP5-AS1	miR-204-5p	0.049	3.47E-01

lncRNA	mRNA	R value	*p* value
MALAT1	PPIA	− 0.165	1.30E−03**^a^
LINC01232	PPIA	0.123^a^	1.68E−02*^a^
DHRS4-AS1	PPIA	− 0.018	7.32E−01
OIP5-AS1	PPIA	− 0.417	3.33E−17***^a^

^a^These results are statistically significant.

*p value < 0.05; **p value < 0.01; ***p value < 0.001.

**Figure 6:**
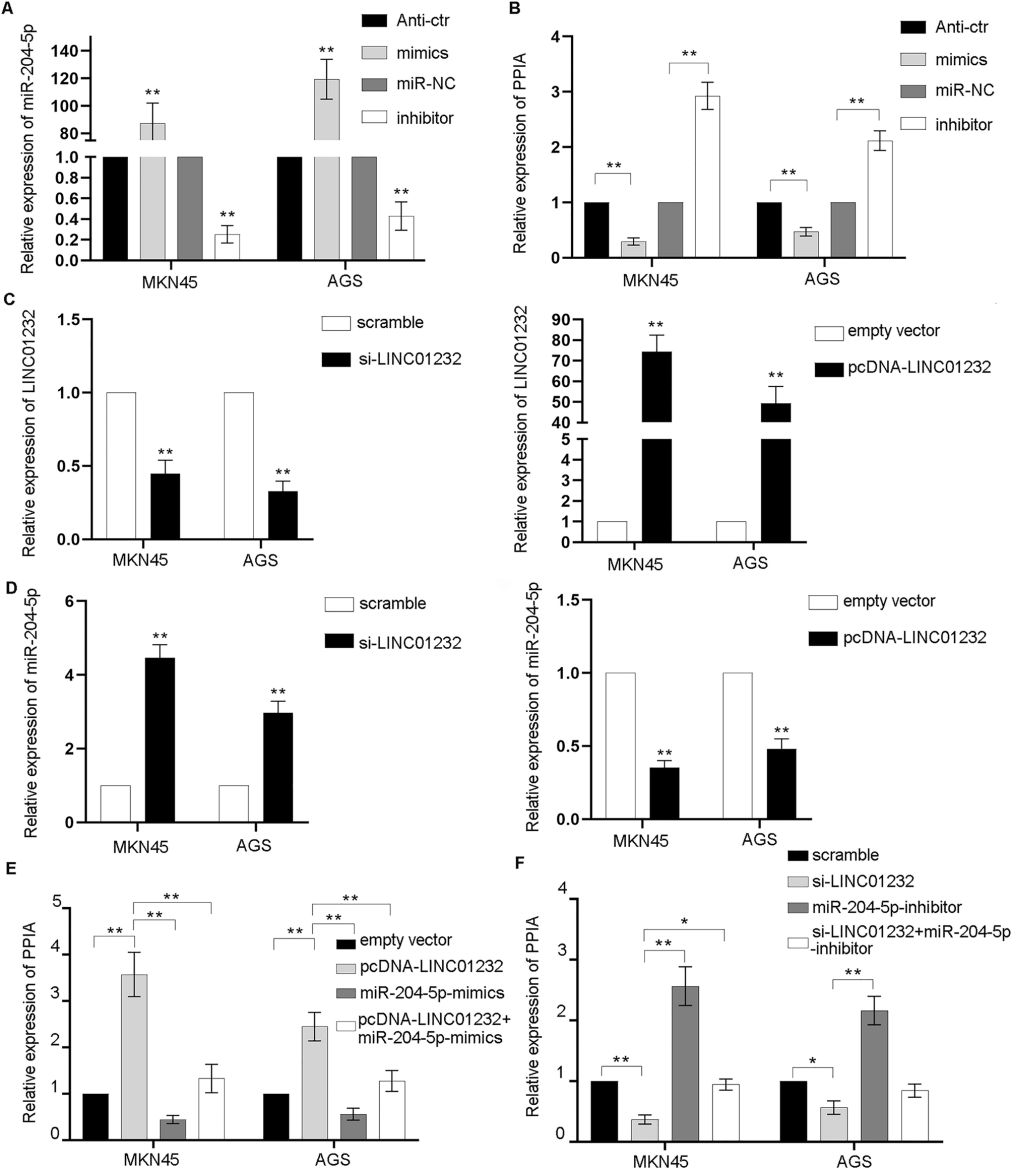
qRT-PCR experimental verification the relationship between LINC01232 and miR-204-5p, PPIA. (**A**) The relative expression level of miR-204-5p in MKN45 and AGS cells after transfected with miR-204-5p mimics, miR-204-5p inhibitor, or control miRNA. **p < 0.01. (**B**) The relative expression level of PPIA in MKN45 and AGS cells after transfected with miR-204-5p mimics, miR-204-5p inhibitor, or control miRNA. **p < 0.01. (**C**) The expression of LINC01232 was obviously decreased or increased in MKN45 and AGS cells after transfection of si-LINC01232 or pcDNA-LINC01232 compared to control group. **p < 0.01. (**D**) The expression of miR-204-5p was significantly upregulated or downregulated in MKN45 and AGS cells after transfected with si-LINC01232 or pcDNA-LINC01232 compared with control group. **p < 0.01. (**E**) PPIA mRNA expression level in MKN45 and AGS cells after overexpressed of LINC01234 and/or miR-204-5p. **p < 0.01. (**F**) PPIA mRNA level in MKN45 and AGS cells after knockdown of LINC01232 and/or inhibition of miR-204-5p. *p < 0.05; **p < 0.01.

In order to verify this conclusion, we overexpressed or knocked down miR-204-5p and LINC01232 in MKN45 and AGS cells. Quantitative real time polymerase chain reaction (qRT-PCR) was used to measure the transfection efficiency. As shown in Fig. 6A, transfection of miR-204-5p mimics or inhibotor significantly upregulated or knocked down miR-204-5p expression in MKN45 and AGS cells compared with the control group (*p* < 0.01). LINC01232 was obviously downregulated or overexpressed in MKN45 and AGS cells after transfected with si-LINC01232 and pcDNA-LINC01232 compared to control group (*p* < 0.01, Fig. 6C). To determine if miR-204-5p could regulate the PPIA expression in GC. We examined PPIA mRNA level when miR-204-5p was overexpressed or inhibited in MKN45 and AGS cells. The result exhibited that PPIA were obviously decreased or increased by miR-204-5p overexpression or inhibition (*p* < 0.01, Fig. 6B). To explore the lncRNA-miRNA-mRNA (ceRNA) network, we also measured miR-204-5p expression after LINC01232 was overexpressed or knocked down in MKN45 and AGS cells. The data suggested that miR-204-5p were obviously upregulated or downregulated by LINC01232 inhibition or overexpression (*p* < 0.01, Fig. 6D). We also conducted rescue experiments to clarify this pathway and our data showed that up-regulation of LINC01232 increased PPIA expression obviously, while overexpression of miR-204-5p reduced PPIA expression dramatically compared with the control group (*p* < 0.01, Fig. 6E). Similarly, the suppression of PPIA expression level induced by si-LINC01232 was effectively reversed by the miR-204-5p inhibitor compared to the control group (Fig. 6F). Taken together, our results suggested that LINC01232/miRNA-204-5p/PPIA network might act as a potential biological pathway in GC.

### Negative correlation between PPIA and ICI in GC

**Figure 7:**
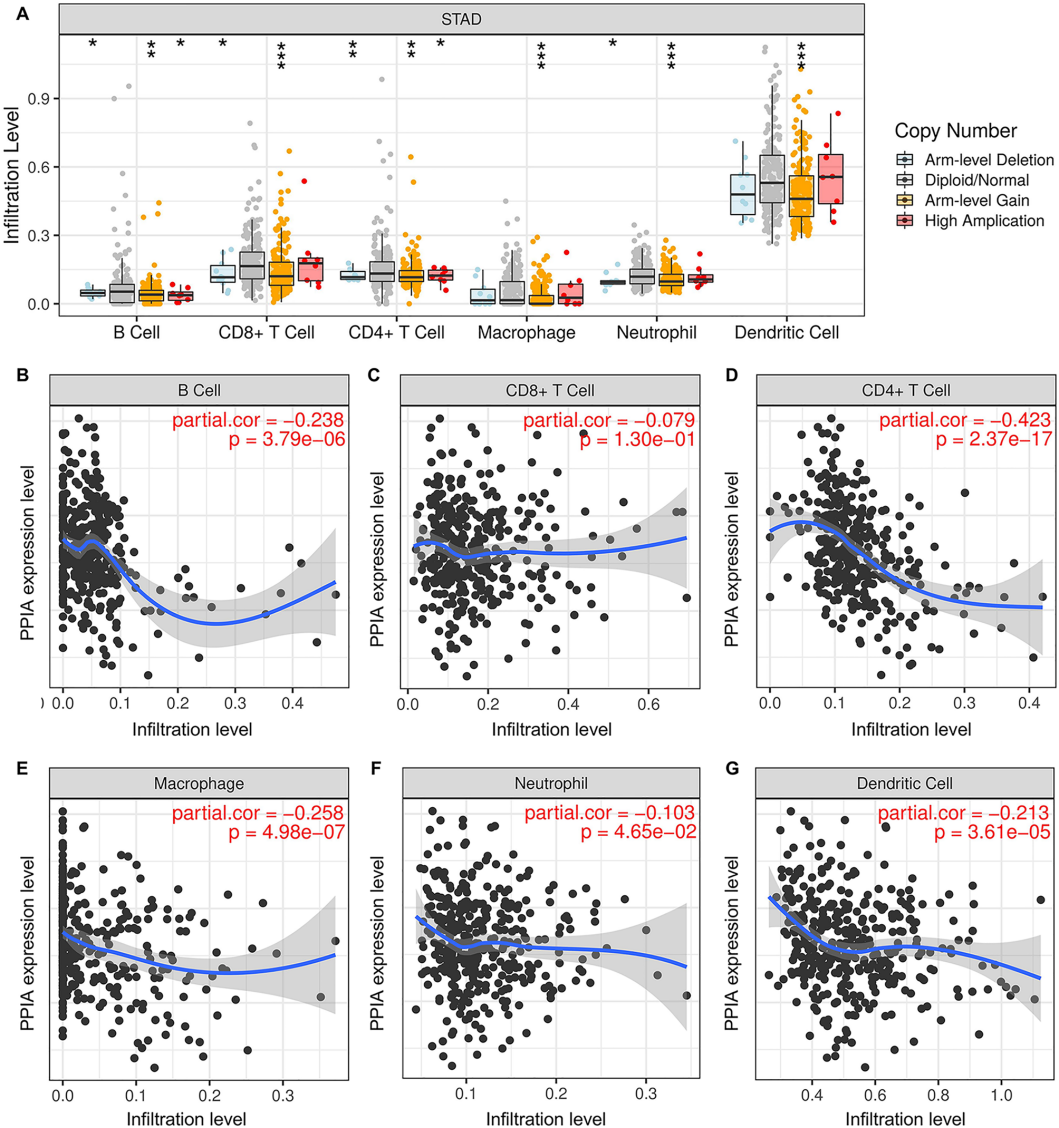
The correlation between PPIA and ICI in GC. (**A**) The infiltration levels of different immune cells under multiple distinct copy numbers of PPIA in GC. (**B**) The relationship between PPIA expression and B cell (**B**), CD8^+^ T cell (**C**), CD4^+^ T cell (**D**), macrophage (**E**), neutrophil (**F**) and dendritic cell (**G**) infiltration levels in GC.

PPIA encoded a member of the peptidyl-prolyl cis–trans isomerase family and had a function of cyclosporin A-mediated immunosuppression, indicating that PPIA may play a pivotal role in functionality of the immune system. Thus, TIMER database was applied to discuss the relationship between PPIA expression and ICI level (Fig. 7A). In B cell and CD4^+^ T cell, Arm-level deletion group, Arm-level gain group and High amplication group displayed negative changes in the ICI levels of PPIA compared to diploid/normal group. In CD8^+^ T cell and neutrophil cell, arm-level deletion group and arm-level gain group also showed negative changes in the ICI levels of PPIA compared to normal control. In macrophage cell and dendritic cell, only arm-level gain group exhibited an negative change in the ICI level of PPIA compared to normal controls. However, the correlation coefficients between PPIA expression and ICI were small. We deduced that PPIA expression might negatively correlated to the ICI levels of CD8^+^ T cell, CD4^+^ T cell, B cell, dendritic cell, macrophage and neutrophil in GC (Fig. 7B–G).

Correlation analysis between PPIA and immune cell biomarkers or immune checkpoints in GC.

**Figure 8:**
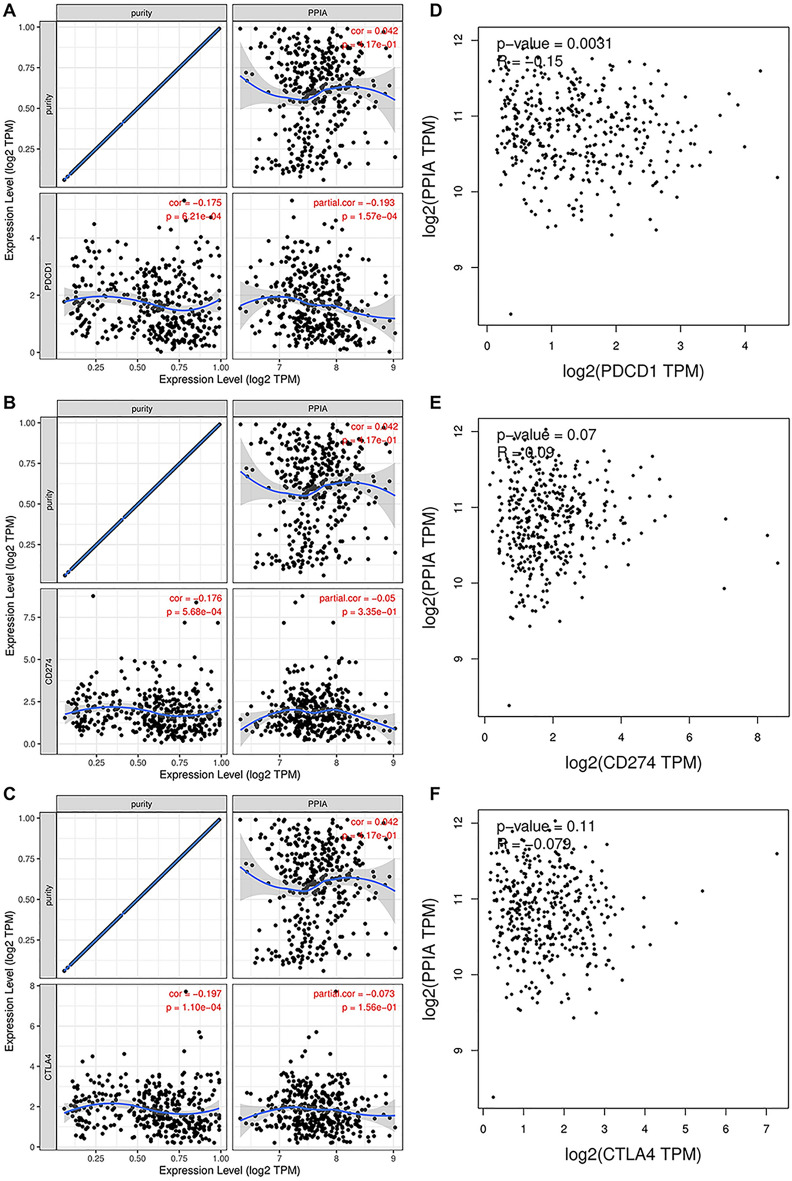
The correlation between PPIA and CTLA-4, PD-L1, PD-1 expression in GC. The correlations between PPIA expression and PD-1 (**A**), PD-L1 (**B**) and CTLA-4 (**C**) were analyzed by Spearman’s method using TIMER database. The correlation analyses between PPIA expression and PD-1 (**D**), PD-L1 (**E**) and CTLA-4 (**F**) were performed using GEPIA database.

To fully explain the mechanism of PPIA in tumor immunity in GC, GEPIA database was used to confirm the relationship between PPIA expression and biomarkers of immune cells. As presented in Table 2, PPIA was negatively correlated with B cell’s biomarkers (CD79A and CD19), CD4^+^ T cell’s biomarker (CD4), CD8^+^ T cell’s biomarker (CD8A), M2 macrophage’s biomarker (MS4A4A), neutrophil’s biomarkers (CCR7 and ITGAM) and dendritic cell’s biomarkers (HLA-DPB1, CD1C, ITGAX and NRP1). These results also suggested that PPIA was negatively related to ICI in GC. It is well known that cytotoxic T-lymphocyte-associated protein 4(CTLA-4) and programmed death 1 (PD1)/programmed cell death-Ligand 1(PD-L1) have been demonstrated to be important checkpoints that block anti-tumor immune responses. Previous studies and this paper confirmed PPIA could act as an oncogene in various tumors and GC. To further analyze the correlation between PPIA expression and immune checkpoints in GC, TIMER database was used to perform the correlation analysis. PPIA expression was negatively related to PD1, PD-L1, and CTLA-4 in GC (Fig. 8A–C), however, the correlation coefficients between PPIA expression and immune cells were small. Our results showed that only the correlation between PD1 and PPIA possessed a significant *P* value (Fig. 8D–F). Overall, the results suggest that PPIA might be participated in anti-tumor immunity progression in GC.

**Table 2:** Correlation analysis between PPIA and biomarkers of immune cells in GC performed by GEPIA database.

Immune cell	Biomarker	R value	*p* value
B cell	CD19	− 0.37	7.1E−15***
	CD79A	− 0.39	1.6E−16***
CD8^+^ T cell	CD8A	− 0.19	1.5E−04***
	CD8B	− 0.079	0.11
CD4^+^ T cell	CD4	− 0.16	1.2E−03**
M1 macrophage	NOS2	0.034	0.5
	IRF5	− 0.017	0.73
	PTGS2	− 0.035	0.48
M2 macrophage	CD163	− 0.054	0.28
	VSIG4	− 0.056	0.26
	MS4A4A	− 0.12	0.012*
Neutrophil	CEACAM8	− 0.049	0.32
	ITGAM	− 0.23	3.4E−06***
	CCR7	− -0.36	1.4E−13***
Dendritic cell	HLA-DPB1	− 0.12	0.014*
	HLA-DQB1	− 0.029	0.56
	HLA-DRA	− 0.032	0.52
	HLA-DPA1	− 0.066	0.18
	CD1C	− 0.36	8.3E−14***
	NRP1	− 0.2	3.4E−05***
	ITGAX	− 0.19	1.5E−04***

*p value < 0.05; **p value < 0.01; ***p value < 0.001.

## Discussion

Until now, GC still is a fatal cancer with a high mortality rate. Patients with GC tend to have a poor clinical prognosis due to lack of effective treatment. Therefore, the development and establishment of novel early diagnostic and therapeutic targets for GC are urgently needed. Previous research has shown that PPIA plays a vital role in oncogenesis and appears in many cancer networks^16^, including GC. Nevertheless, the specific causal mechanism of PPIA in GC remains to be fully elucidated.

Herein, we reported the high expression of PPIA in pan-cancer, which were partially consistent with Yu’s findings^26^. We confirmed that PPIA was markedly upregulated in GC via multiple databases, and proved that overexpression of PPIA was related to poor prognosis. We also investigated the expression level of PPIA in 53 GC patients. Our results showed PPIA was significantly overexpressed in GC tissues compared with the corresponding normal tissues. However, there were no significant difference between PPIA expression and GC prognosis. This result might be related to short follow-up time period (The median follow-up time in our study was 26.6 months). We also performed the relevance between PPIA expression and clinical parameters in 53 GC patients and the result were inconsistent with TCGA samples. The differences might be partly due to a smaller number of cases in our study and the study worthy of further evaluation. Jia et al.^32^ reported that the correlation between PPIA and lymph node metastasis in lung adenocarcinoma, constructed a reliable risk score model and provided valuable biomarkers for lung cancer patients. We also found that high expression of PPIA had a close relationship to clinical parameters of GC patients, including tumor grade, cancer stage, nodal metastasis status, gender, age and so on. Analysis of these data, in combination with our results, suggest that PPIA may play a tumor promoting role in GC.

Numerous studies have demonstrated that genetic alteration and epigenetic modifications could regulate gene expression at different levels. Jin and co-workers reported that oxidative DNA damage was correlated with inflammation and cancer. SBS18 is one type of oxidative DNA damage which could be caused by reactive oxygen species and commonly appeared in gastric adenocarcinomas^33^. Herein, the genetic alteration status of PPIA in GC was analyzed, and the results demonstrated that amplification was the only alteration type in GC. Furthermore, no mutation site of PPIA was observed in GC and patients in PPIA alteration group did not show better prognosis in OS, DFS and PFS. Epigenetic alterations, especially DNA methylation, also have a major impact in cancer progression. Padmanabhan et al.^34^ indicated that cystathionine beta-synthase enzyme was a highly recurrent target of epigenetic silencing and could act as a modifier of aberrant DNA methylation, which provided potential new therapy of GC. Hsu et al.^35^ found that METTL4 regulated 6 mA expression in mammalian tumor cells and contribute to cancer metastasis by activating multiple metastasis-inducing genes. However, in our results, the aberrant expression of PPIA was not obviously associated with DNA methylation, suggesting genetic alteration and DNA methylation of PPIA might have a negligible effect in the process of gastric carcinogenesis.

Histone modification, DNA methylation and non-coding RNAs are important component of epigenetic modifications. Numerous studies have proved that non-coding RNAs also play crucial roles in tumor progression^36,37^. However, little is known about the upstream miRNAs of PPIA in GC. To identify the potential upstream regulatory miRNAs of PPIA, StarBase 3.0 was used to predict PPIA-related miRNAs. Finally, three miRNAs, including let-7c-5p, let-7e-5p and miRNA-204-5p, were chosen as candidate upstream miRNAs of PPIA in GC. Among these miRNAs, let-7c-5p was obviously downregulated and negatively related to SEMA3F in hepatocellular carcinoma and its upregulation was positively correlated with GC prognosis^38^. Let-7c-5p also inhibited PBX3 expression and suppressed the epithelial-mesenchymal transition and prevented the malignant progression of laryngeal squamous cell carcinoma^39^. Camila and colleagues reported that let-7e-5p was overexpressed in colorectal cancer and could act as a non-invasive predict marker for the diagnosis of colorectal cancer^40^. miRNA-204-5p was finally chosen as the upstream miRNA of PPIA after comprehensive consideration of expression, correlation and survival analyses. miRNA-204-5p is obviously deregulated in GC and restoration of miRNA-204-5p expression facilitated GC cell apoptosis^41^. Another study also revealed that miRNA-204-5p significantly inhibited the expression of lncSLCO1C1 and suppressed GC progression by preventing cell growth and promoting DNA damage^42^.

According to the corresponding regulatory mechanism, the upstream lncRNA of miRNA-204-5p and PPIA could play an oncogene role in GC. Forty-four suitable lncRNAs were detected by StarBase 3.0 database and LINC01232 was selected as the final candidate target correlated with miRNA-204-5p and PPIA. Interestingly, previous studies confirmed that LINC01232 could play an oncogenic role in various types of malignancies including GC^43–46^. Meng et al.^47^ revealed that linc01232 was obviously upregulated in pancreatic cancer and high levels of linc01232 expression were significantly correlated with poor prognosis. Our result demonstrated that miRNA-204-5p silencing or overexpression could affect PPIA expression. Moreover, LINC01232 silencing or overexpression also affected miRNA-204-5p expression and rescue experiment confirmed that PPIA expression level regulated by LINC01232 was dramaticly reversed by the miR-204-5p. Therefore, LINC01232/miRNA-204-5p/PPIA axis might act as a potential biological pathway in GC.

Tumor infiltrated immune cells were a vital component of tumor microenvironment and involved in tumorigenesis and progression^48–50^. However, the role of PPIA in modulating the immune system in GC is still elusive. Our results proved that PPIA was negatively related to ICI in GC. Interestingly, these results were generally consistent with observations reported previously^51^. However, opposite results were observed in hepatocellular carcinoma^26^. Thus, the association between PPIA expression and immune checkpoints was analyzed through GEPIA database. This study found that PPIA expression was negatively related to CTLA-4, PD-L1, and PD1 in GC. Altogether, our results revealed that PPIA might be participated in anti-tumor immunity progression and mediated immune evasion in GC. In summary, PPIA was upregulated in various kinds of human cancer, including GC, and associated with poor prognosis in GC. Abnormal expression of PPIA was considered unrelated to gene mutation and DNA methylation. We confirmed the upstream regulators of PPIA and established a LINC10232/miRNA-204-5p/PPIA axis in GC. Our results also demonstrated that PPIA was negatively correlated to ICI and immune checkpoints in GC. Nevertheless, these results require further experimental validation.

## Methods

### TCGA data analysis

PPIA mRNA expression in 33 cancer types was retrieved from TCGA database^52^. R package limma was utilized to explore the differential expression between tumor and normal tissues.

### GEPIA

GEPIA (http://gepia.cancer-pku.cn/index.html)^53^ is an online web tool for gene expression analysis according to GTEx and TCGA data. In this study, the differential expression analysis of PPIA in GC and the prognostic evaluation of upstream lncRNAs were conducted using GEPIA.

### UALCAN

UALCAN (http://ualcan.path.uab.edu/)^54^ is a web tool for providing depth analysis of transcription data based on MET500 and TCGA data. We explored the expression of PPIA and its association with different clinicopathological parameters (tumor grade, cancer stage, nodal metastasis status, TP53 mutation status, race, gender, age, *H. pylori* infection status and histological subtypes) of GC.

### Kaplan–Meier (K–M) plotter analysis

The K–M plotter (http://kmplot.com/analysis/)^55^ is a web tool that provides gene survival analysis in various cancer types. The GC specimens were assigned to low and high PPIA expression groups, the survival analysis including progression-free survival (PFS), post-progression survival (PPS) and overall survival (OS) of PPIA in GC were conducted. Survival analysis for the upstream miRNAs of PPIA in GC was also described using K–M plotter.

### Functional enrichment analysis (GO and KEGG) and GSEA

GO and KEGG analyses^56^ were conducted to study the enrichment function of PPIA based on the differentially expressed genes. GSEA^57^ was carried out to elucidate potential mechanisms of PPIA using the clusterProfiler in R software.

### cBioPortal

cBioPortal (http://www.cbioportal.org/)^58^ is a friendly online tool to explore the genomic alteration frequency and type of PPIA in GC. The disease-free survival (DFS), disease-specific survival (DSS), progression-survival and OS of GC related to PPIA alteration status were also determined using cBioPortal.

### Methylation and expression analysis of PPIA

UALCAN (http://ualcan.path.uab.edu/)^54^ and DiseaseMeth version 3.0 (http://diseasemeth.edbc.org)^59^ were utilized to explore the methylation levels of PPIA between GC and corresponding normal tissues.

### Starbase database analysis

StarBase 3.0 (http://starbase.sysu.edu.cn/)^60^ can be applied to explore miRNA-related research. StarBase 3.0 contains multiple tools, including RNA22, PITA, microT, miRmap, PicTar, miRanda and TargetScan, to estimate the upstream miRNAs and lncRNAs of PPIA. Only the lncRNAs and miRNAs demonstrated on more than two tools could select as candidates of PPIA. The expression correlations among miRNA-204-5p, LINC01232 and PPIA were conducted by starBase 3.0.

### TIMER

TIMER (https://cistrome.shinyapps.io/timer/)^61^ is an open web portal which focuses on the analysis of ICI. TIMER was employed to discuss the relationship between PPIA expression and ICI level or immune checkpoint expression level.

### Clinical samples

53 paired of GC tissues and corresponding normal tissues were collected from the First Affiliated Hospital of Zhengzhou University between 2020 and 2021. All samples were confirmed with histology and no patient underwent chemotherapy or radiotherapy before surgical. All samples were immediately frozen in liquid nitrogen and stored at − 80 °C after surgical removal. The study was approved by the Ethics Committee of the First Affiliated Hospital of Zhengzhou University and all patients signed informed consent forms (ZBMT001). All methods were performed in accordance with the relevant guidelines and regulations.

### Cell culture

Three human gastric cancer cell lines (MKN45, AGS and HGC27) and a normal gastric epithelium cell line (GES-1) were obtained from Chinese Academy of Sciences, Shanghai Institutes for Cell Resource Center. Cells were seeded into culture dishes containing Dulbecco’s minimal essential medium (DMEM), 10% serum, pen-Icillin (100 U/ml), and streptomycin (0.1 mg/ml) at 37 °C with 5% CO_2_.

### Cell transfection

MKN45, AGS and HGC27 and GES-1 cells were seeded into 6-well culture plates and were transfected with siRNAs [miR-204-5p mimics, miR-204-5p inhibitor, LINC01232siRNAs and scrambled negative control siRNA (si-NC)] (Genechem, Shanghai) and plasmid vectors using Lipofectamine 3000 (Invitrogen). The nucleotide sequences of siRNAs and plasmid vectors were shown in Supplementary Table 2. Cells were collected for qRT-PCR 48 h after transfection.

### RNA extraction and qRT-PCR

We used TRIzol reagent (Invitrogen) to extract total RNA based on the manufacturer's instructions, PrimeScript RT Reagent Kit (TaKaRa) and SYBR Premix Ex Taq (Takara) were used to reverse transcription and real-time PCR analysis according to the manufacturer's instructions. Primers of PPIA, GAPDH, miR-204-5p, U6, LINC01232 were obtained from Genechem (Shanghai) and the primers were listed in Supplementary Table 1. ABI 7500 real-time PCR system was utilized to perform the qRT-PCR experiments and 2^−∆∆Ct^ method was used to data analysis.

### Statistical analysis

The significant difference between 2 groups was compared by Student’s *t* test. Chisquare test was used to analyze the relationship between PPIA and the clinical parameters factors of GC patients. Kaplan–Meier analysis was utilized to analyze the correlation between PPIA expression and the overall survival of GC patients with log rank test used for comparison. *P* < 0.05 was deemed statistically significant.

### Ethics approval

Our study was approved by the ethics committee of The First Affiliated Hospital of Zhengzhou University.

### Supplementary Information

Supplementary Information 1.

Supplementary Information 2.

Supplementary Information 3.

## Data Availability

The data used and analyzed in this article are available from the corresponding author on reasonable request.
