# Supplementary material for: Bioinformatics analysis and experimental validation of tumorigenic role of PPIA in gastric cancer
Source: Sci Rep. 2023 Nov 5;13:19116. doi: 10.1038/s41598-023-46508-y (PMC10625987; doi:10.1038/s41598-023-46508-y)
Supplement: Supplementary file 1 — Supplementary Information 1. [file 41598_2023_46508_MOESM1_ESM.pdf]

Figure S1

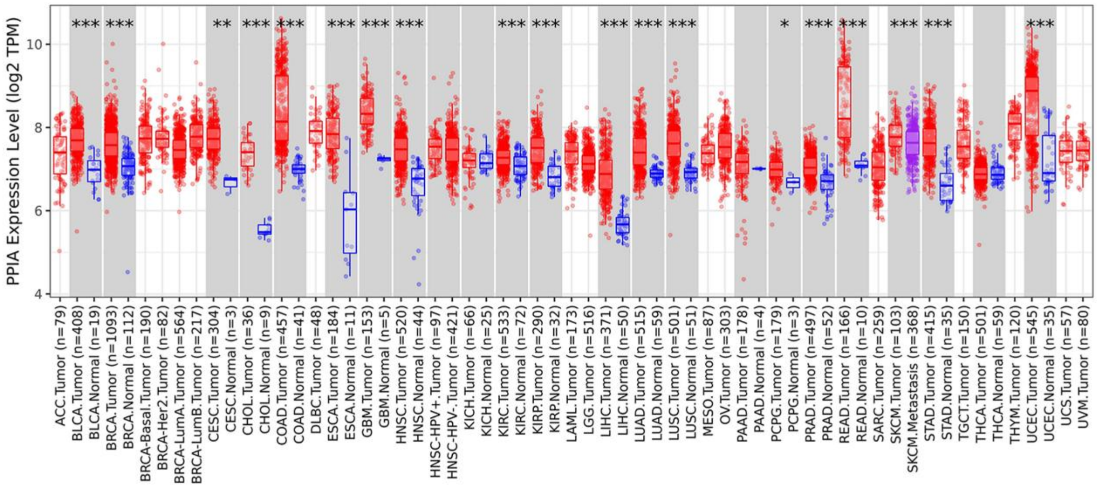

Figure S1 Expression levels of PPIA in 33 human cancer types based on TIMER database.

Figure S2

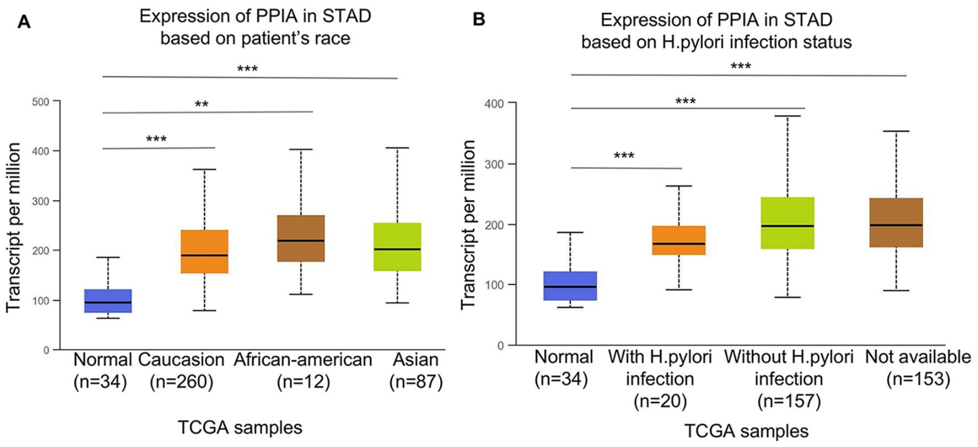

Figure S2 The relationship between PPIA expression and different clinical parameters in GC according to UALCAN database. The clinical parameters include patient's race (A), H. pylori infection status (B) . **p<0.01; ***p<0.001.

Figure S3

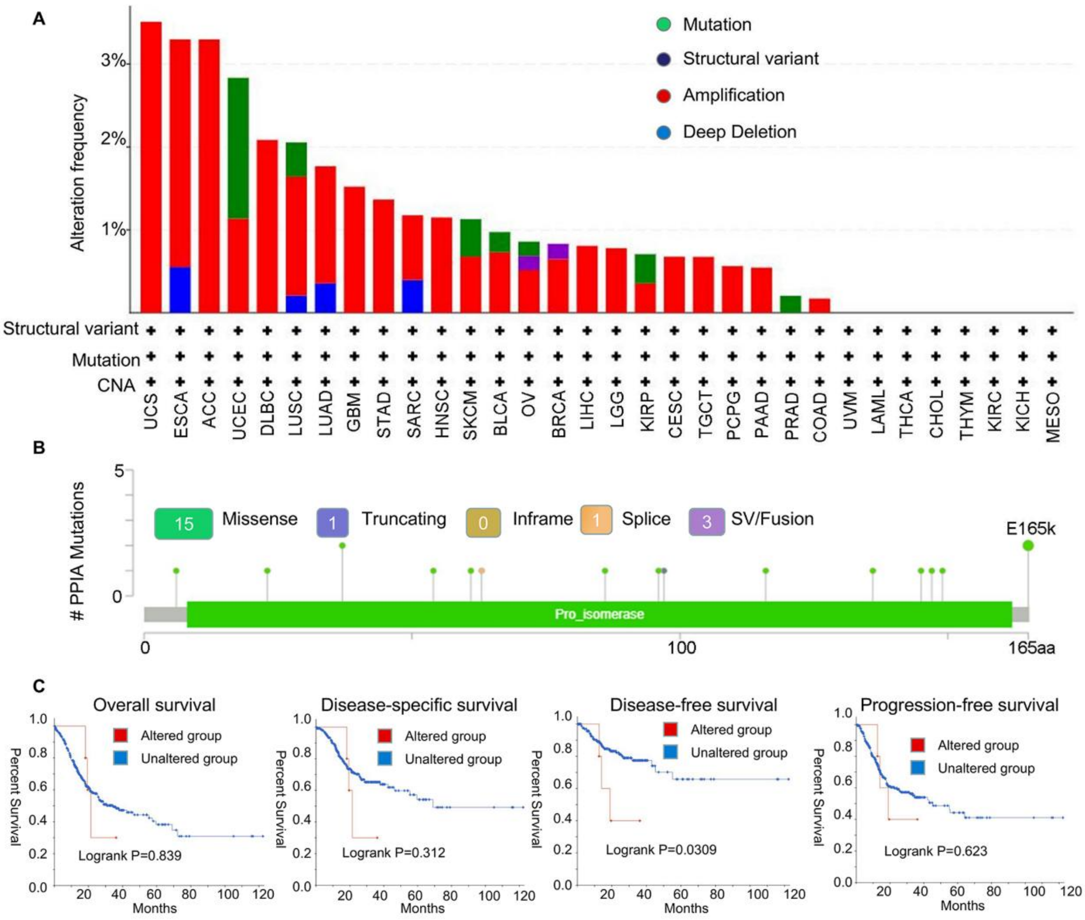

Figure S3 Mutation status of PPIA in pan-cancer according to TCGA database. Genetic alteration status of PPIA in multiple types of cancers were explored according to cBioPortal web tool. The mutation type (A) and mutation site (B) of PPIA in pan-cancer. The correlation between PPIA mutation status and survival prognosis of GC was displayed using cBioPortal database (C).

Figure S4

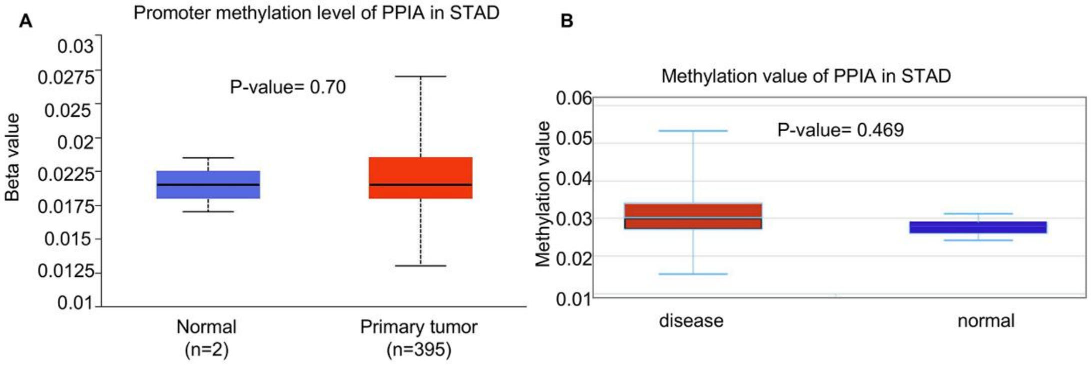

Figure S4. Methylation analysis of PPIA in GC. (A) Methylation analysis of PPIA was displayed using UALCAN database. (B) Methylation analysis of PPIA was explored based on DiseaseMeth version 3.0.

Figure S5

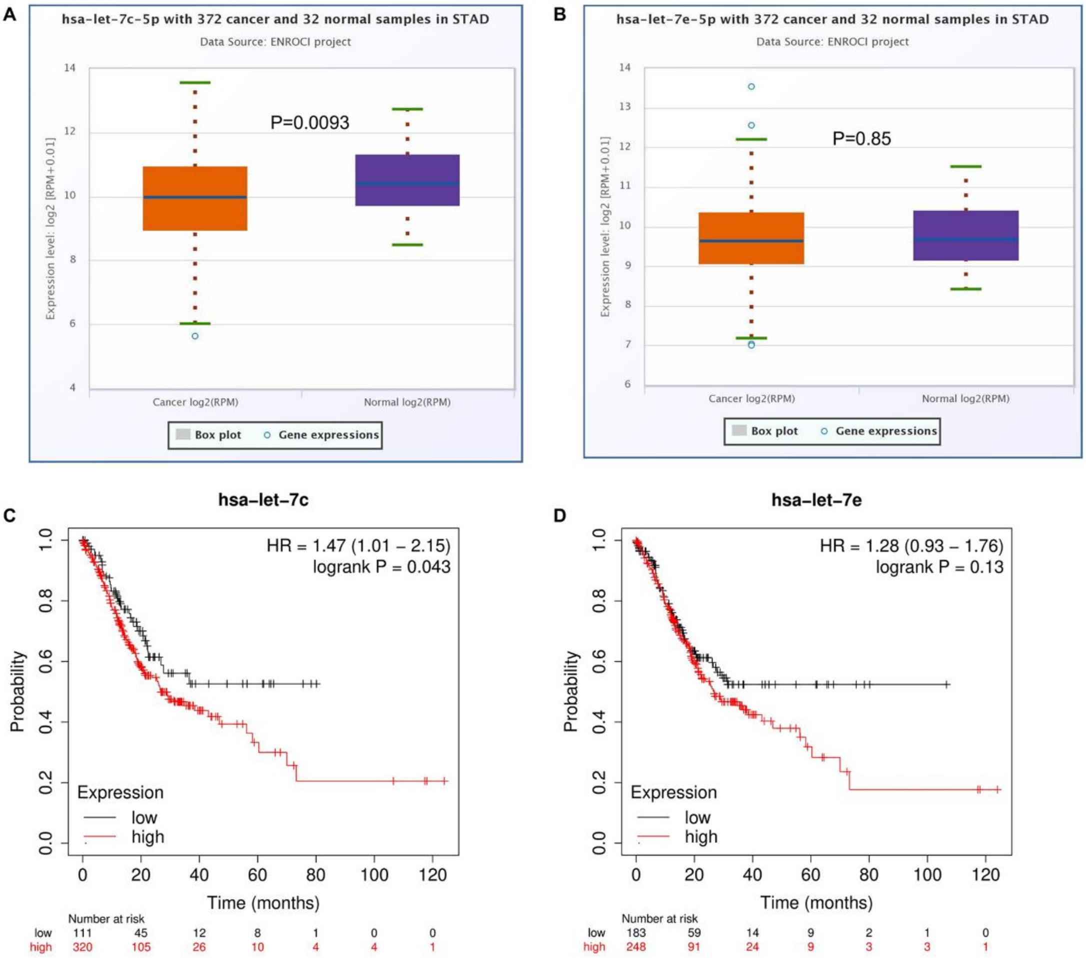

Figure S5 Expression levels of let-7c-5p (A) and let-7e-5p (B) in GC confirmed using starBase 3.0 database. The prognostic values of let-7c-5p (C) and let-7e-5p (D) in GC were analyzed using K-M plotter.

Figure S6

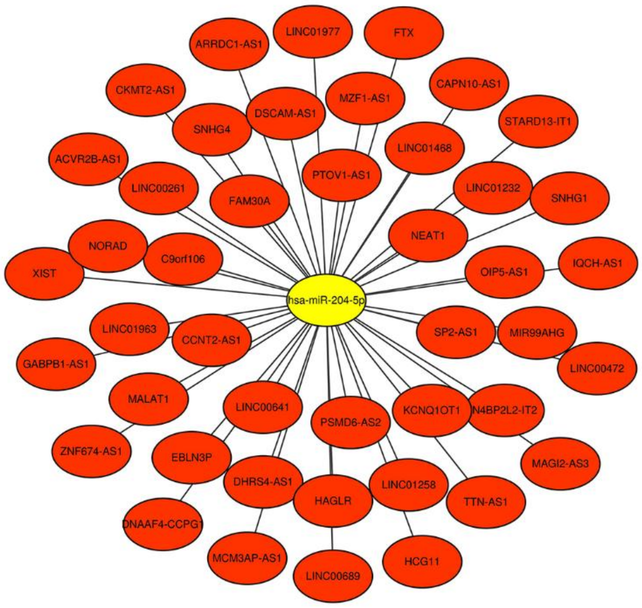

Figure S6 LncRNA-miRNA-204-5p interaction networks constructed by Cytoscape_3.2.1(https://cytoscape.org/) software.
