# Supplementary material for: Bioinformatics analysis and experimental validation of tumorigenic role of PPIA in gastric cancer
Source: Sci Rep. 2023 Nov 5;13:19116. doi: 10.1038/s41598-023-46508-y (PMC10625987; doi:10.1038/s41598-023-46508-y)
Supplement: Supplementary file 2 — Supplementary Information 2. [file 41598_2023_46508_MOESM2_ESM.docx]

Supplementary Table 1 Statistics of PPIA expression and clinical parameters

| clinical parameters | Comparison | Statistical significance (P value) |
| --- | --- | --- |
| individual cancer stages |  |  |
|  | Normal vs Stage 1 | 6.04869999999424E-05 |
|  | Normal vs Stage 2 | 1.62447832963153E-12 |
|  | Normal vs Stage 3 | <1E-12 |
|  | Normal vs Stage 4 | 4.74139949524499E-10 |
|  | Stage 1 vs Stage 2 | 2.602400E-01 |
|  | Stage 1 vs Stage 3 | 3.110200E-01 |
|  | Stage 1 vs Stage 4 | 6.752600E-01 |
|  | Stage 2 vs Stage 3 | 2.851400E-01 |
|  | Stage 2 vs Stage 4 | 2.731000E-01 |
|  | Stage 3 vs Stage 4 | 5.411600E-01 |
| patient’s gender |  |  |
|  | Normal vs Male | <1E-12 |
|  | Normal vs Female | <1E-12 |
|  | Male vs Female | 5.231600E-01 |
| patient’s age(Y) |  |  |
|  | Normal vs Age (21-40Y) | 4.528400E-02 |
|  | Normal vs Age (41-60Y) | 1.62436730732907E-12 |
|  | Normal vs Age (61-80Y) | 1.62447832963153E-12 |
|  | Normal vs Age (81-100Y) | 1.6251444634463E-12 |
|  | Age (21-40Y) vs Age (41-60Y) | 6.220000E-01 |
|  | Age (21-40Y) vs Age (61-80Y) | 9.377800E-01 |
|  | Age (21-40Y) vs Age (81-100Y) | 8.491800E-01 |
|  | Age (41-60Y) vs Age (61-80Y) | 6.580100E-02 |
|  | Age (41-60Y) vs Age (81-100Y) | 2.269200E-01 |
|  | Age (61-80Y) vs Age (81-100Y) | 8.549800E-01 |
| tumor grade |  |  |
|  | Normal vs Grade 1 | 5.79549996881923E-09 |
|  | Normal vs Grade 2 | <1E-12 |
|  | Normal vs Grade 3 | <1E-12 |
|  | Grade 1 vs Grade 2 | 1.875690E-02 |
|  | Grade 1 vs Grade 3 | <1E-12 |
|  | Grade 2 vs Grade 3 | 1.562840E-01 |
| nodal matastasis status |  |  |
|  | Normal vs N0 | <1E-12 |
|  | Normal vs N1 | <1E-12 |
|  | Normal vs N2 | <1E-12 |
|  | Normal vs N3 | 1.62447832963153E-12 |
|  | N0 vs N1 | 7.655000E-01 |
|  | N0 vs N2 | 6.776000E-01 |
|  | N0 vs N3 | 5.101000E-01 |
|  | N1 vs N2 | 5.263600E-01 |
| clinical parameters | Comparison | Statistical significance (P value) |
| nodal matastasis status |  |  |
|  | N1 vs N3 | 7.005000E-01 |
|  | N2 vs N3 | 3.518800E-01 |
| TP 53 mutation status |  |  |
|  | Normal vs TP53 Mutant | 1.62447832963153E-12 |
|  | Normal vs TP53 NonMutant | 1.62447832963153E-12 |
|  | TP53 Mutant vs TP53 NonMutant | 7.936800E-03 |
| patient’s race |  |  |
|  | Normal vs Caucasian | <1E-12 |
|  | Normal vs AfricanAmerican | 1.084480E-03 |
|  | Normal vs Asian | <1E-12 |
|  | Caucasian vs AfricanAmerican | 1.833420E-01 |
|  | Caucasian vs Asian | 8.028200E-02 |
|  | AfricanAmerican vs Asian | 2.287000E-01 |
| H.pylori infection status |  |  |
|  | Normal vs Tumors (with H.pylori infection status) | 2.99630000000217E-06 |
|  | Normal vs Tumors (without H.pylori infection status) | <1E-12 |
|  | Normal vs Tumors (Not available) | <1E-12 |
|  | Tumors (with H.pylori infection status) vs Tumors (without H.pylori infection status) | 1.53477E-01 |
|  | Tumors (with H.pylori infection status) vs Tumors (Not available) | 1.74102E-01 |
|  | Tumors (without H.pylori infection status) vs Tumors (Not available) | 7.5784E-01 |
