# Supplementary material for: Bioinformatics analysis and experimental validation of tumorigenic role of PPIA in gastric cancer
Source: Sci Rep. 2023 Nov 5;13:19116. doi: 10.1038/s41598-023-46508-y (PMC10625987; doi:10.1038/s41598-023-46508-y)
Supplement: Supplementary file 3 — Supplementary Information 3. [file 41598_2023_46508_MOESM3_ESM.docx]

Supplementary Table 2 Sequences of siRNAs and primers

| Names | Sequences |
| --- | --- |
| qPCR primers |  |
| GAPDH forward | 5ʹ-GGGAGCCAAAAGGGTCAT-3′ |
| GAPDH reverse | 5ʹ-GAGTCCTTCCACGATACCAA-3′ |
| U6 forward | 5ʹ-CTCGCTTCGGCAGCACA-3′ |
| U6 reverse | 5ʹ-AACGCTTCACGAATTTGCGT-3′ |
| PPIA forward | 5ʹ-ACCGCCGAGGAAAACCGTGTA-3′ |
| PPIA reverse | 5ʹ-TGCTGTCTTTGGCACCTTGTCTGC-3′ |
| miR-204-5p forward | 5ʹ-ACACTCCAGCTGGGTTCCCTTTGTCATCCTAT-3′ |
| miR-204-5p reverse | 5ʹ-CTCAACTGGTGTCGTGGA-3′ |
| LINC01232 forward | 5ʹ-AGGATGCGCCTAAGAAAGGG-3′ |
| LINC01232 reverse | 5ʹ-CCGGGGGATTGAGGAAACAT-3′ |
| Sequence of siRNAs |  |
| miR-204-5p mimics | 5ʹ-CACACTGGACTAGTGGATCCGCCTTGTTAAGTGCTCGCTTC-3′ |
| miR-204-5p inhibitor | 5ʹ-AGGCATAGGATGACAAAGGGAA-3′ |
| microRNA NC | 5ʹ-CAGUACUUUUGUGUAGUACAA-3′ |
| miRNA-anti-control | 5ʹ-TTCTCCGAACGTGTCACGT-3′ |
| Si-LINC01232 | 5ʹ-GCATAGGCGTGTGACCTTTGT-3′ |
| pcDNA-LINC01232 | 5ʹ-GTGGATCCGAGCTCGGTACCAATTTTATAAAACCTTGAAATC-3′ |
| LINC01232-scramble | 5ʹ-TTCTCCGAACGTGTCACGT-3′ |
| LINC01232-empty vector | 5ʹ-UUCUCCGAACGUGUCACGUTT-3′ |
